# Nitrate inhibition of N_2_ fixation and its effect on micronutrient accumulation in shoots of soybean (*Glycine max* L. Merr.), Bambara groundnut (*Vigna subterranea* L. Vedc) and Kersting’s groundnut (*Macrotyloma geocarpum Harms.*)

**DOI:** 10.1007/s13199-017-0531-2

**Published:** 2017-12-15

**Authors:** Glory Chinonye Mbah, Felix Dapare Dakora

**Affiliations:** 1 Department of Crop Sciences, Tshwane University of Technology, Pretoria, 0001 South Africa; 2 Department of Chemistry, Arcadia Campus, Tshwane University of Technology, Private Bag X680, 175 Nelson Mandela Drive, Pretoria, 0001 South Africa

**Keywords:** Legumes, 5 mM NO_3_^−^, Rhizobial inoculation, Trace element accumulation, Inhibition of nodulation

## Abstract

Although nitrate is known to inhibit nodulation and N_2_ fixation in symbiotic legumes, little is known about its effect on the uptake and accumulation of trace elements such as Fe, Zn, Mn and Cu. The aim of this study was to evaluate the effect of 5 mM NO_3_^−^ supply, either with or without rhizobial inoculation, on nodulation, nodule functioning and micronutrient levels in the shoots of soybean (*Glycine max* L.Merr.), Bambara groundnut (*Vigna subterranea* L. Vedc) and Kersting’s groundnut (*Macrotyloma geocarpum* Harm). The results showed reduction in plant growth, nodule formation and nodule dry matter by the supply of 5 mM NO_3_^−^ to inoculated seedlings of all three species. Nitrate inhibition respectively caused 1.2, 1.4, and 1.5-fold decrease in nodule number per plant in Bambara groundnut, soybean and Kersting’s bean, which resulted in 2.3, 3.3 and 4.5-fold reduction in nodule dry weight of the test species (in that order). The application of 5 mM NO_3_^−^ to soybean plants also resulted in 2.5, 4.0 and 5.4-fold decrease in shoot accumulation of Fe, Zn and Mn, respectively, when compared to the purely symbiotic control plants. Furthermore, we observed 1.3, 1.8 and 1.3-fold decreases in the concentration of Zn, Mn and Cu in shoots of inoculated Bambara groundnut with NO_3_^−^ supply, levels lower than those found in soybean. With Kersting’s groundnut, shoot concentration of Fe, Zn and Cu were higher with the application of 5 mM NO_3_^−^ to inoculated plants when compared to the purely symbiotic treatment, which was opposite to soybean. But pure NO_3_^−^feeding of this species respectively resulted in 2.0, 1.4 and 1.3-fold decreases in Fe, Zn and Cu relative to inoculated NO_3_^−^-fed plants. Clearly, NO_3_^−^ supply to landraces/genotypes of the three legume species did not only inhibit nodule formation and functioning, it also reduced shoot micronutrient levels in soybean and Bambara groundnut, but not Kersting’s bean.

## Introduction

Mineral nutrition is important for growth of plants, especially nodulated legumes due to the high demand for nutrients by N_2_-fixing bacteroids in root nodules (Israel 1987; Udvardi and Poole 2013). Recent studies on cowpea have shown a strong relationship between N_2_ fixation and mineral accumulation (Belane et al. 2014). However, several factors can inhibit nodulation and N_2_ fixation in legumes, which in turn affects mineral accumulation in symbiotic legumes. The enzyme nitrogenase, which reduces N_2_ to NH_3,_ is O_2_-labile and therefore denatures when the O_2_ concentration is high (Dakora and Atkins 1989). Mineral N (nitrate and ammonium) is also a potent inhibitor of nodule formation and N_2_ fixation (Streeter and Wong 1988; Ayisi et al. 2000). Nitrate, in particular, can combine with leghaemoglobin to form nitrosyl-leghaemoglobin, which reduces O_2_ supply to N_2_-fixing bacteroids (Appleby 1984). Waterlogging of nodulated legumes can also result in reduced N_2_ fixation due to low O_2_ (Minchin and Summerfield 1976), though some legumes (e.g. *Psorolea, Sesbania, Neptunia* and *Aeschynomene* species) are reported to grow, nodulate and fix N_2_ under low O_2_ conditions as typically found in wetlands and flooded soils (Kanu and Dakora 2017; James et al. 2001).

However, if indeed mineral accumulation in nodulated legumes is symbiotically-linked (Belane et al. 2014), then inhibition of N_2_ fixation in root nodules by any of the factors mentioned should alter the uptake and accumulation of minerals in legume organs. Furthermore, imposing an inhibition on nodule functioning ought to reduce N_2_ fixation and hence mineral accumulation. Although in this study we measured both micronutrients and macronutrients, the focus is on trace elements because about 263 million African people are suffering from micronutrient deficiency. The aim of this study was to use nitrate as an inhibitor of N_2_ fixation to test whether applying this solute to nodulated legumes can reduce nodulation and N_2_ fixation, and thus decrease mineral uptake and accumulation in plant organs.

## Materials and methods

### Description of study sites

The study was conducted in a naturally-lit glasshouse located at the Tshwane University of Technology, Pretoria, South Africa during the summer months of October to December 2015. Day and night temperatures were not controlled but average maximum and minimum temperatures were 15 and 28 °C respectively.

### Source of seeds

The soybean genotypes used in this study included PAN1614 from PANNAR Seed Company in South Africa, as well as TGx1830-20E and TGx2001-25DM from the International Institute for Tropical Agriculture, Mozambique. The Kersting’s groundnut/Kersting’s bean landraces (Belane, Boli, Heng Milk Mottled, Puffeun, Heng Red Mottled) were sourced from Ghana while the Bambara landraces (Red, Brianbeck, Cream, Black and Mottled) were obtained from South Africa and Ghana.

### Planting

Surface-sterilized seeds of landraces/genotypes of the three legume species (soybean, Kersting’s and Bambara groundnuts) were planted in autoclaved sand contained in 3-L capacity pots. The experimental treatments included i) rhizobial inoculation, ii) rhizobial inoculation +5 mM KNO_3_supply, and iii) 5 mM KNO_3_-feeding. Bambara and Kersting’s groundnuts were inoculated using peat-based commercial inoculant of *Bradyrhizobium* strain CB756 which is a known commercial inoculant for Bambara and Kersting’s groundnuts (Stimuplant, Pretoria). In contrast, soybean was inoculated with *Bradyrhizobium japonicum* strain WB74, its compatible microsymbiont used for commercial soybean production in South Africa. Each treatment was replicated 5 times. Three surface-sterilized seeds were planted per pot and later thinned to one plant per pot at 7 days after germination. The inoculated plants were watered with full-strength N-free nutrient solution (Broughton and Dilworth 1971). Nitrate-fed plants were watered with 5 mM KNO_3_ solution. Watering was done twice a week but the frequency increased as the demand for water also increased with plant growth.

### Plant harvest and processing

Plant harvest was based on the time of flowering of each species. Soybean and Kersting’s groundnut plants were harvested at 44 days after emergence (DAE), while the Bambara groundnut plants were harvested at 63 DAE. Each plant sample was separated into shoots and roots and kept in separate labelled paper bags. In the laboratory, the shoots were oven-dried (60 °C) for 48 h, weighed and ground into fine powder (0.85 mm) for analysis of minerals.

### Nodulation studies

At harvest, nodulated roots were taken to the laboratory and carefully washed to remove sand. Nodules were then plucked, counted, and oven-dried (60 °C) for 72 h for assessing dry matter per plant.

### Determination of micro- and macronutrients in plant shoots

The determination of micronutrients in plant shoots was carried out at the Institute for Plant Sciences, Department of Agriculture, Western Cape, South Africa, following the procedure outlined in the Agri-laboratory Association of Southern Africa handbook. Each shoot sample (2.0 g) was ashed overnight in a crucible at 60 °C and 5 mL of 1:1 HCl added and left to stand in an oven overnight at 60 °C. The solution was filtered, and distilled water added to make it up to 40 mL. The samples were then analyzed for both major and trace elements using inductively coupled plasma-mass spectrometry (ICP-MS).

### Correlation analysis

Ground plant shoots were analyzed for both micro- and macronutrients, and correlation analysis performed between shoot mineral concentrations and symbiotic parameters (shoot biomass, nodule number and nodule dry weights) in order to establish physiological relationships (if any) between symbiosis and mineral accumulation.

### Statistical analysis

All the collected data were tested for normal distribution before being subjected to analysis of variance to compare treatment means using STATISTICA program (version 10) and GENSTAT (11th edition). A 2-way ANOVA was used to analyze the data set, and where there were significant differences, the Duncan’s multiple range test was used to separate the means (*p* ≤ 0.05).

## Results

### Effect of genotype/landrace on plant growth and nodulation

Of the three soybean genotypes studied, PAN 1614 and TGx2001-25DM produced significantly more nodule numbers than TGx1830-20E (Table 1). As a result, those two genotypes also produced much greater nodule dry matter (Table 1). Shoot biomass followed the same trend.

**Table 1:** Nodulation, plant growth and micronutrient accumulation in shoots of soybean genotypes grown under glasshouse conditions

	Nodule number	Nodule dry matter	Shoot dry matter	Fe	Zn	Mn	Cu
	plant^−1^	mg. plant^−1^	g. plant^−1^	μg. g^−1^			
Genotypes							
PAN 1614	54a	130a	3.58a	212.5b	87.0b	115.1ab	6.3b
TGx1830-20E	30b	60b	2.84b	354.5a	90.2b	131.8a	9.1a
TGx2001-25DM	43a	120a	3.14ab	118.1c	177.7a	88.3b	5.8b
Treatment							
Inoc	74a	230a	3.79a	381.4a	197.4a	240.9a	5.1b
Inoc +5 mM NO_3_^−^	54b	70b	2.46b	153.6b	108.2b	50b	6.6b
5 mM NO_3_^−^	0c	0c	3.32a	150.1b	49.4c	44.3b	9.3a
F-statistics							
Genotype (G)	21.65***	36.81***	4.57*	327.53***	20.35***	5.27*	5.9*
Treatment (T)	212.03***	460.28***	14.84***	264.03***	42.66***	136.17***	8.37**
G x T	6.46**	10.37***	4.29*	114.51***	16.28***	18.1***	3.36*
CV (%)	18.5	16.2	16.4	9.6	28.9	25.7	31

There were significant variations in nodulation and plant growth among landraces of Kersting’s groundnut (Table 2). Landrace Belane produced the highest number of nodules per plant (34 in number), while Heng Red Mottled produced the lowest, only 17 nodules per plant. The number of nodules were similar for the other landraces. However, the highest nodule dry weight was produced by landrace Puffeun, and the least by Heng Red Mottled landrace. The Belane landrace produced the least shoot biomass, with the other landraces producing similar shoot biomass (Table 2).

**Table 2:** Nodulation, plant growth and micronutrient accumulation in shoots of Kersting’s groundnut landraces grown under glass house conditions at the Tshwane University of Technology, South Africa

	Nodule number	Nodule dry matter	Shoot dry matter	Fe	Zn	Cu
	plant^−1^	mg.plant^−1^	g.plant^−1^	μg.g^−1^		
Landraces						
Belane	34.0a	20.0bc	0.90b	430.0a	139.63a	8.64a
Boli	27.0b	30.0b	1.51a	204.5bc	75.54b	7.41b
Heng milk mottled	26.0b	20.0bc	1.60a	113.8d	65.74c	3.84d
Puffeun	28.0b	100.0a	1.52a	212.9b	59.2 cd	4.23c
Heng red mottled	17.0c	10.0c	1.80a	171.4c	55.31d	3.53d
Treatment						
Inoc	47.0a	90.0a	1.92a	224.7b	68.30b	4.58c
Inoc + NO_3_^−^	32.0b	20.0b	1.29b	301.0a	98.96a	6.77a
5 mM NO_3_^−^	0c	0c	1.19b	153.9c	69.99b	5.24b
F-statistics						
Landraces (L)	5063***	95.32***	1051***	102.29***	202.58***	305.99***
Treatment (T)	196.24***	32.55***	23.92***	63.79***	83.23***	118.54***
L x T	177.22***	25.59***	3.96**	46.84***	115.98***	133.8***
CV (%)	5	49.2	21.3	15.7	9.2	7.2

Bambara groundnut landraces also differed in plant growth and root nodulation (Table 3). The Mottled landrace showed greater nodulation (30 nodules plant^−1^), followed by Brianbeck (28 nodules plant^−1^) and then the Red landrace (24 nodules plant^−1^). The Black and Cream landraces produced the least number of nodules (Table 3). However, nodule dry matter was similar for all landraces, except Cream which produced significantly lower nodule dry weight (Table 3). Shoot biomass was markedly higher in the Mottled landrace, followed by Brianbeck, with the Cream and Red landraces producing the lowest shoot biomass (Table 3).

**Table 3:** Nodulation, plant growth and nutrient accumulation in shoots of Bambara groundnut landraces grown under glasshouse conditions

	Nodule number	Nodule dry matter	Shoot dry matter	Fe	Zn	Mn	Cu
	plant^−1^	mg.plant^−1^	g.plant^−1^	μg.g^−1^			
Landraces							
Black	21d	100a	1.61bc	256.2b	80.9b	65.5b	6.3b
Mottled	30a	110a	1.94a	294.0a	80.5b	47.6c	12.4a
Brianbeck	28b	110a	1.75ab	251.5b	66.8c	65.6b	6.5b
Cream	20d	80b	1.45c	143.9c	68.0c	60.5b	4.0c
Red	24c	110a	1.51c	234.9b	90.6a	73.5a	7.1b
Treatment							
Inoc	41a	210a	1.73a	214.5b	85.51a	89.40a	8.43a
Inoc + NO_3_^−^	34b	90	1.7a	244.4a	79.29a	49.79b	6.65b
5 mM NO_3_^−^	0c	0c	1.53b	249.5a	67.18b	48.42b	6.67b
F-statistics							
Landraces (L)	26.67***	8.8***	8.59***	37.67***	9.89***	19.83***	82.1***
Treatment (T)	1192.25***	946.01***	4.29*	7.19**	14.46***	196***	14.91***
L x T	12.79***	5.93***	3.01*	16.76***	19.83***	24.26***	20.38***
CV (%)	9.8	13.1	12.3	11.6	12.3	10.3	14.2

### Effect of nitrate on root nodulation

Supplying 5 mM NO_3_^−^ to inoculated soybean plants depressed nodulation (nodule number and dry weight) relative to inoculated purely symbiotic plants. In fact, it caused 1.4-fold decrease in nodule number, and 3.3-fold decrease in nodule dry weight relative to purely symbiotic plants (Table 1). The 5 mM NO_3_^−^-fed plants (without inoculation) had no root nodules (Table 1).

With Kersting’s groundnut, supplying NO_3_^−^ to inoculated plants also markedly depressed nodulation. The provision of 5 mM NO_3_^−^ reduced nodule number per plant by 1.5-fold and nodule dry weight by 4.5-fold relative to purely symbiotic plants (Table 2). The 5 mM NO_3_^−^-fed plants (without inoculation) produced no root nodules (Table 2).

Nodulation in Bambara groundnut was also inhibited by NO_3_^−^ application. There was a 1.2-fold reduction in nodule number per plant, and 2.3-fold decrease in nodule dry weight per plant when compared to purely symbiotic plants. The 5 mM NO_3_^−^-fed plants (without inoculation) had no nodules on their roots (Table 3).

### Effect of genotype/landrace on micronutrient concentration in shoots

Of the three soybean genotypes tested, TGx1830-20E showed markedly greater levels of Fe, Mn and Cu, while TGx2001-25DM recorded the lowest, except for Zn (Table 1). Kersting’s bean landrace Belane consistently revealed much higher levels of Fe, Zn and Cu in shoots, with Heng red mottled showing the lowest concentrations of Zn and Cu (Table 2). With Bambara groundnut, landrace Mottled exhibited increased levels of Fe and Cu in shoots, while the Red landrace recorded the highest levels of Zn and Mn (Table 3). The Cream landrace generally showed much lower shoot concentration of Fe and Cu.

### Effect of NO_3_^−^-feeding on micronutrient distribution in shoots

Although in soybean there were significant differences in shoot concentration of Fe, Zn, Mn and Cu, NO_3_^−^ supply, either with or without inoculation, consistently reduced shoot levels of Fe, Zn and Mn. Relative to the inoculated, purely symbiotic soybean plants, there was 2.5, 4.0 and 5.4-fold decrease in shoot Fe, Zn and Mn in 5 mM NO_3_^−^-fed plants, and 2.5, 1.8 and 4.8-fold reduction in nodulated NO_3_^−^-fed plants (Table 1).

With Bambara groundnut, shoot concentration of Zn, Mn and Cu was greater in purely symbiotic plants relative to the NO_3_^−^ treatments (Table 3). In fact, there was 1.3, 1.8 and 1.3-fold decrease in the levels of Zn, Mn and Cu with 5 mM NO_3_^−^-feeding, and 1.1, 1.8 and 1.3-fold reduction of Zn, Mn and Cu in plants that were inoculated with *Bradyrhizobium* and treated to 5 mM NO_3_^−^ (Table 3).

With Kersting’s bean, the purely symbiotic plants showed lower levels of Fe, Zn and Cu relative to the other treatments. As a result, the inoculated NO_3_^−^-fed plants revealed 1.9, 1.4 and 1.3-fold increase in Fe, Zn and Cu relative to purely NO_3_^−^-fed plants, and 1.3, 1.4 and 1.5-fold increase in Fe, Zn and Cu when compared to nodulated purely symbiotic plants (Table 2).

Inoculating soybean plants with *Bradyrhizobium japonicum* strain WB74 markedly increased the concentration of Fe, Zn and Mn in shoots when compared to purely NO_3_^−^ -fed plants or the inoculated + NO_3_^−^ treatment (Table 1). In fact, the inoculated + NO_3_^−^ plants also generally accumulated more Fe, Zn and Mn than the purely NO_3_^−^-fed plants (Table 1). However pure NO_3_^−^-feeding accumulated more Cu in soybean shoots than the other two treatments (Table 1). In Kersting’s groundnut landraces, the inoculated + NO_3_^−^ treatment increased the concentration of the micronutrients Fe, Zn and Cu over the sole inoculated and purely NO_3_^−^ fed plants (Table 2).

With Bambara groundnut, bacterial inoculation increased the shoot concentration of Zn, Mn and Cu but decreased that of Fe (Table 3). Pure NO_3_^−^ - feeding and inoculated + NO_3_^−^ plants accumulated more Fe in Bambara groundnut shoots relative to *Bradyrhizobium* inoculation alone (Table 3).

### Genotype x inoculation interaction on nodulation and plant growth

**Fig. 1:**
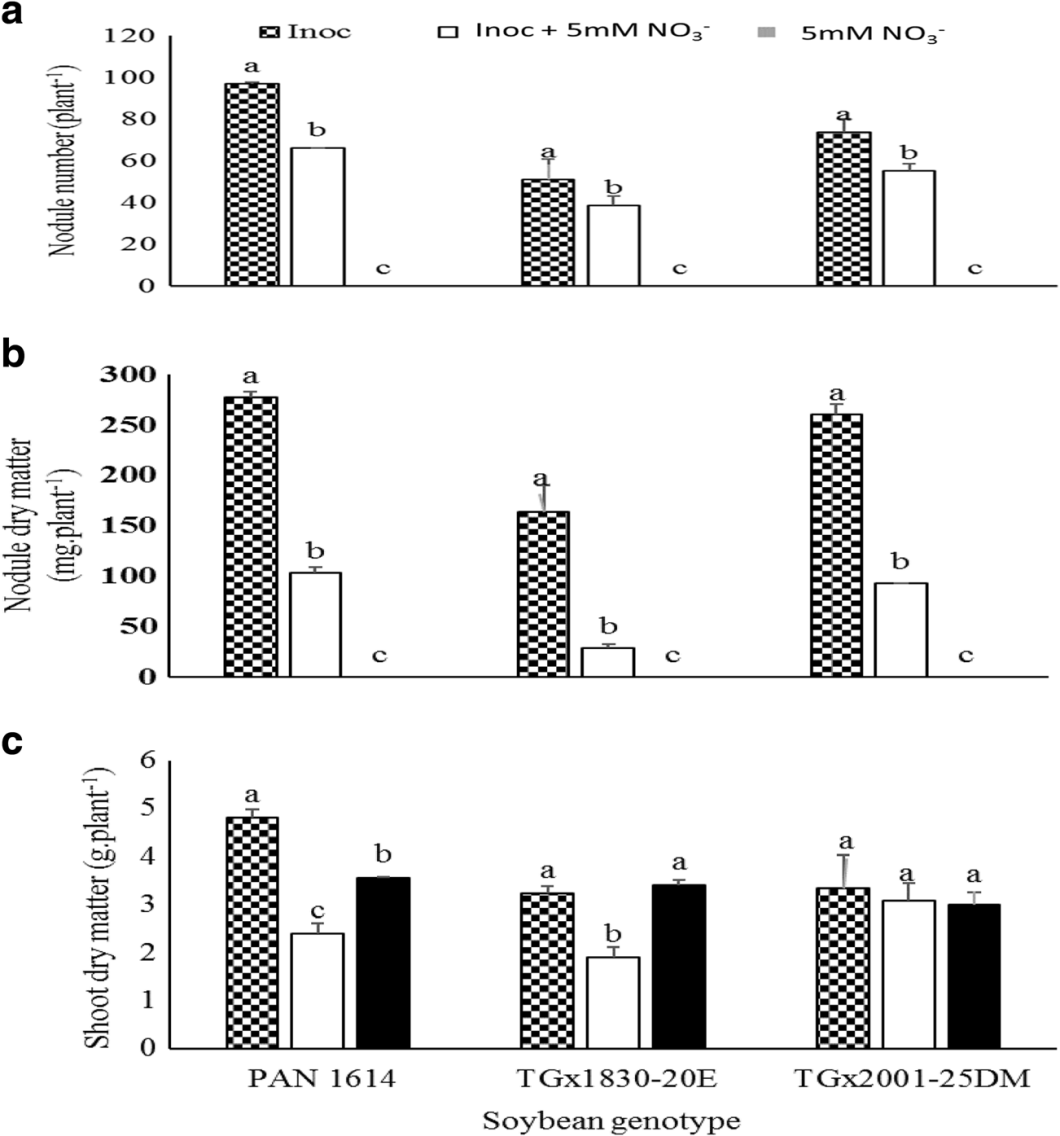
Genotype x treatment interaction on **a** Nodule number **b** Nodule dry matter and **c** Shoot dry matter of soybean genotypes grown in the glasshouse

The genotype x inoculation interaction was significant for nodule number, nodule dry weight and shoot biomass of soybean, Kersting and Bambara groundnuts (Tables 1, 2 and 3). For soybean, *Bradyrhizobium* inoculation alone significantly increased the nodule numbers and nodule dry weight in all three test genotypes, followed by the inoculated + NO_3_^−^ treatment (Fig. 1a and b). There were no nodules on soybean plants receiving only 5 mM NO_3_^−^. For PAN 1614, *Bradyrhizobium* inoculation on its own significantly promoted plant growth and increased shoot biomass, followed by pure NO_3_^−^ feeding and least was inoculated + NO_3_^−^ (Fig. 1c). Plant growth (measured as shoot biomass) was increased equally by bacterial inoculation and NO_3_^−^ feeding of soybean genotype TGx1830-20E while the inoculated + NO_3_^−^ promoted the least plant growth in this genotype (Fig. 1c). With genotype TGx2001-25DM, shoot dry matter was not affected by *Bradyrhizobium* application or NO_3_^−^ feeding (Fig. 1c).

**Fig. 2:**
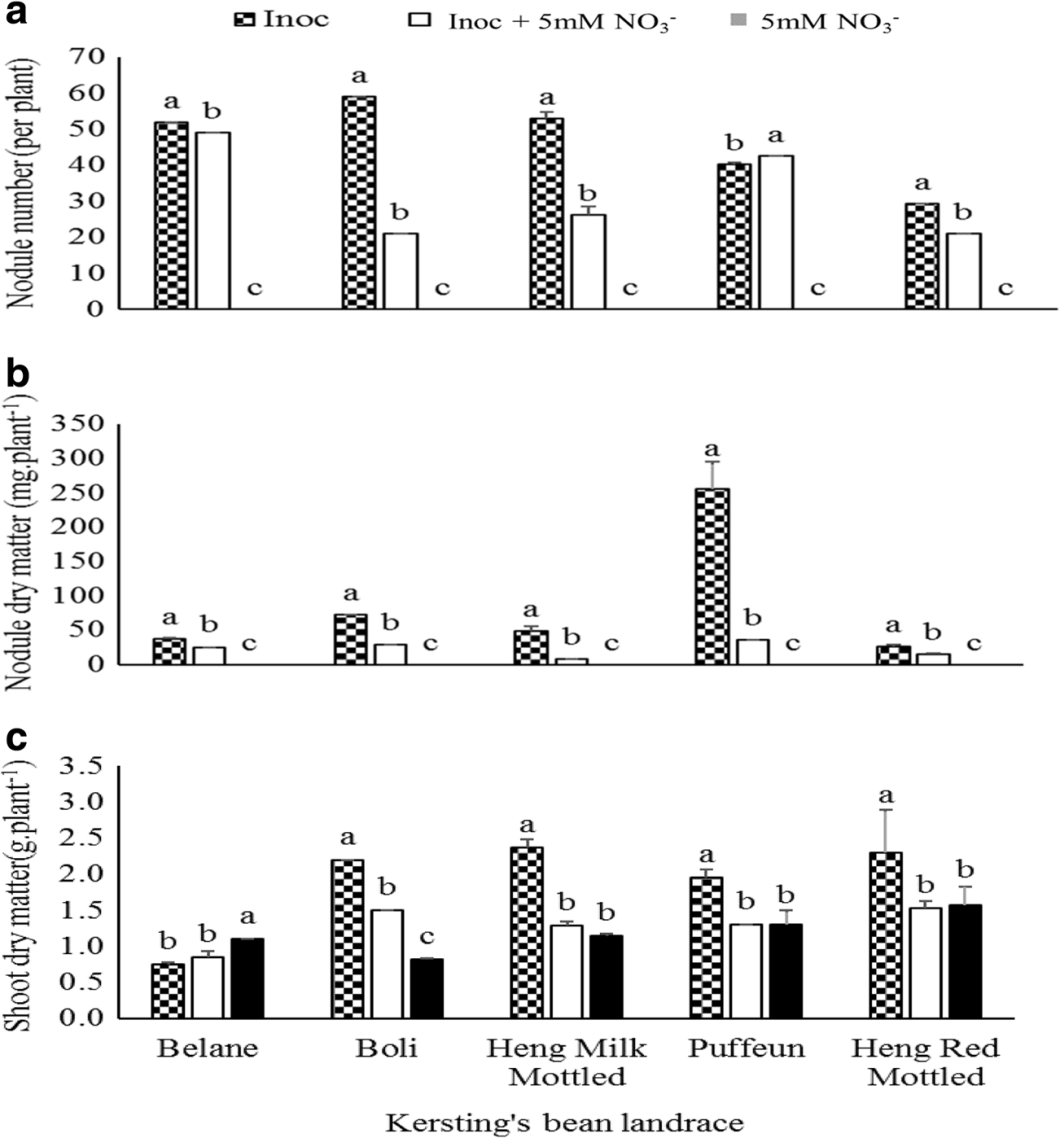
Landrace x treatment interaction on **a** Nodule number **b** Nodule dry matter and **c** Shoot dry matter of Kersting’s bean landraces grown in the glasshouse

As found for soybean, the genotype x inoculation analysis showed increased nodule number (except in the Puffeun landrace) and dry mass in all the Kersting’s landraces when solely inoculated (Fig. 2a and b). Plant growth measured as shoot biomass was also markedly increased in the Boli, Heng Milk Mottled, Puffeun and Heng Red Mottled landraces when solely inoculated with *Bradyrhizobium* over the inoculated + NO_3_^−^ and NO_3_^−^ feeding (Fig. 2c). However, in the Belane landrace, the highest shoot biomass was recorded in the pure NO_3_^−^ fed plants. Relative to the pure NO_3_^−^ fed plants, the inoculated + NO_3_^−^ treatment produced higher shoot biomass in the Boli landrace while in Heng milk mottled, Puffeun and Heng red mottled landrace, similar shoot biomass was produced with both treatments (Fig. 2c).

**Fig. 3:**
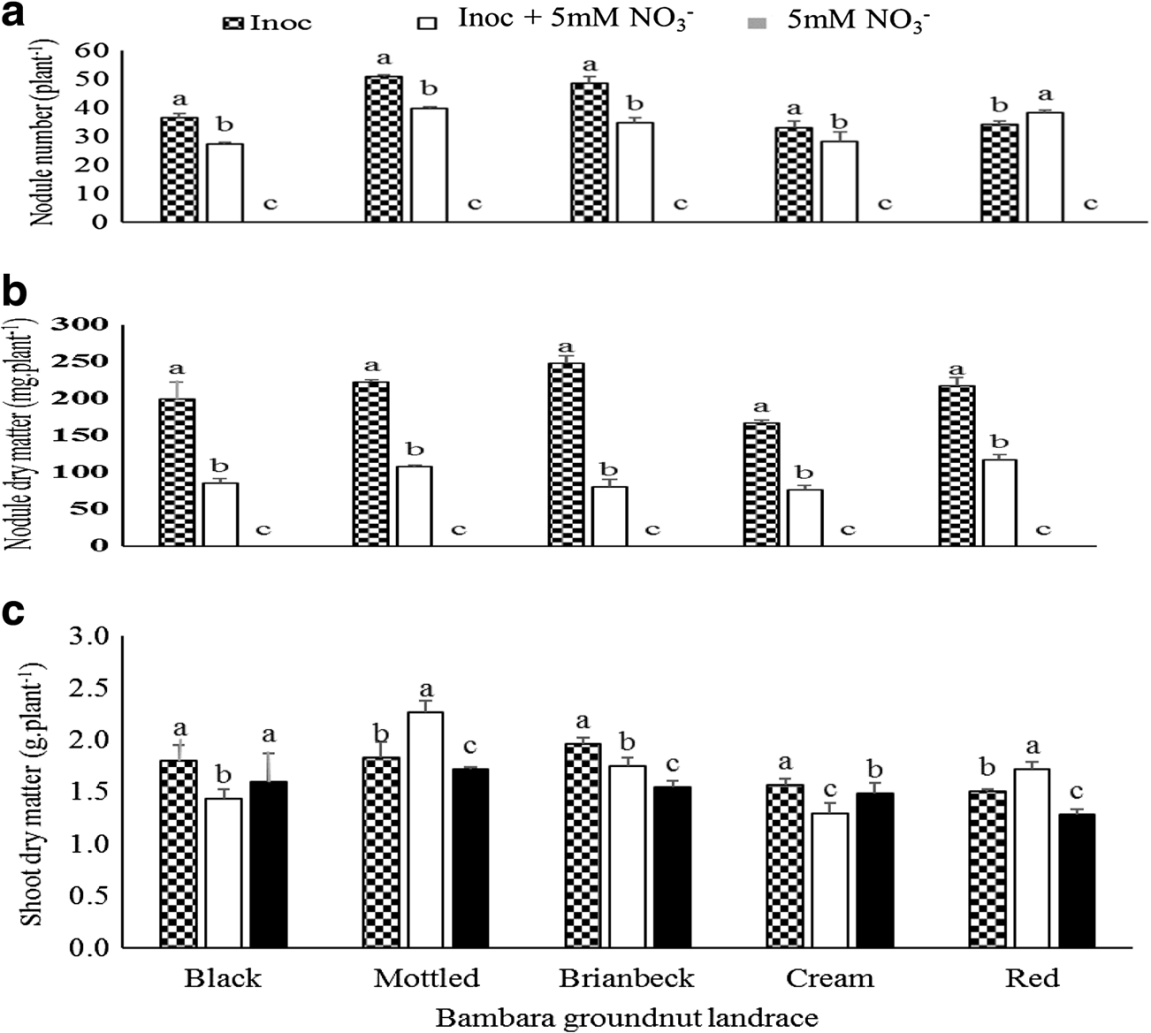
Genotype x treatment interaction on **a** Nodule number **b** Nodule dry matter and **c** Shoot dry matter of Bambara landraces grown in the glasshouse

The genotype x inoculation analysis also showed that *Bradyrhizobium* application to Bambara groundnut significantly increased nodule number (except Red) and nodule dry weight per plant in all five landraces when compared to inoculated + NO_3_^−^ treatment (Fig. 3a and b). There were no nodules on pure NO_3_^−^ fed pants. However, plant growth was differentially regulated by the treatments imposed. As shown in Fig. 3c, bacterial inoculation on its own promoted plant growth in only the Black, Brianbeck and Cream landraces when compared to pure NO_3_^−^ feeding and inoculated + NO_3_^−^. The latter treatment also promoted greater plant growth in only the Mottled and Red landraces (Fig. 3c). Of the five landraces, only Black showed similar and equal shoot growth promotion with NO_3_^−^ feeding and *Bradyrhizobium* inoculation alone (Fig. 3c).

### Genotype x inoculation interaction on micronutrient distribution in plant shoots

**Fig. 4:**
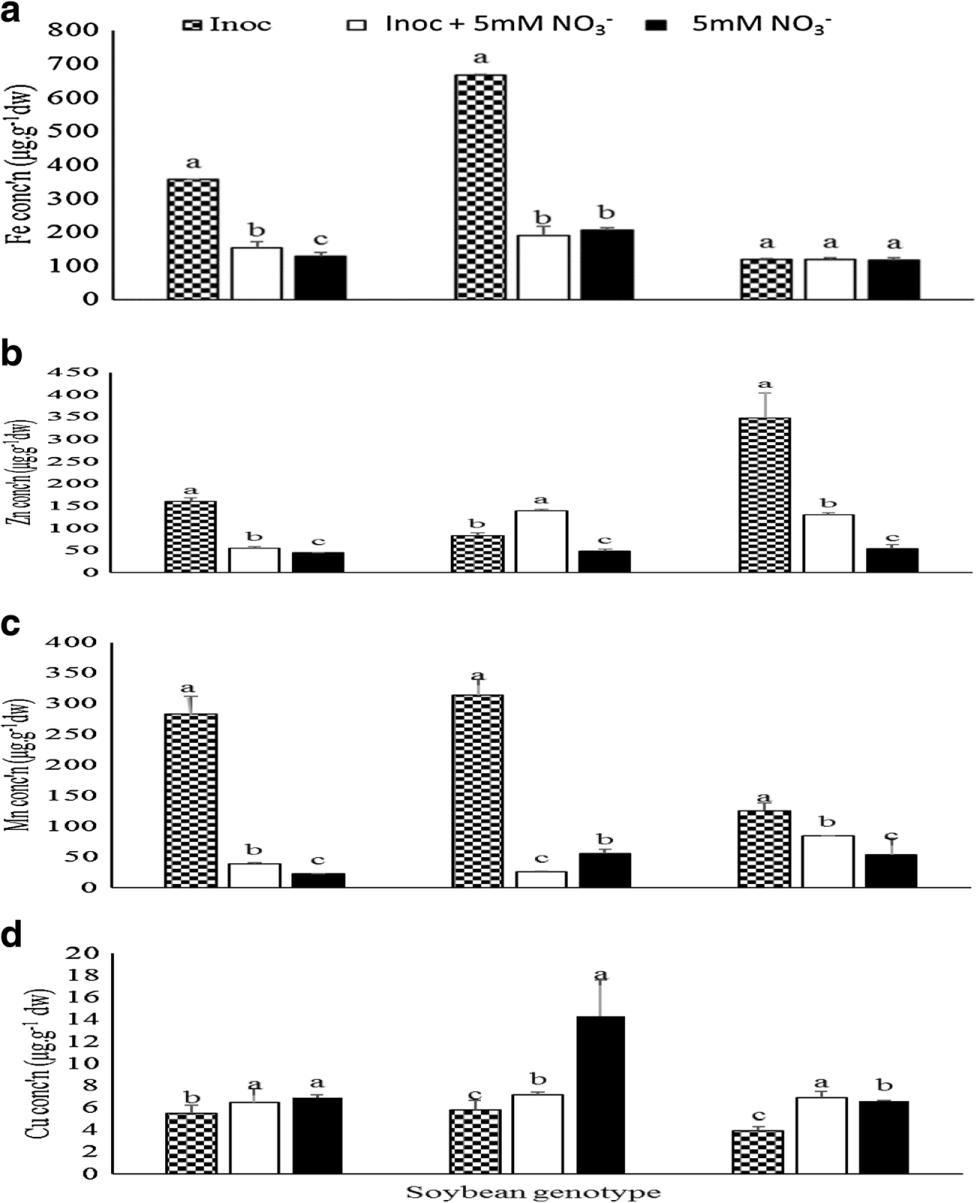
Genotype x treatment interaction on **a** Fe **b** Zn and **c** Mn **d** Cu concentration in shoots of soybean genotypes grown in the glasshouse

The genotype x inoculation interaction was significant for Fe, Zn, Mn and Cu concentrations in shoots of the test genotypes and landraces (Tables 1, 2, and 3). Although shoot Fe concentrations were unaltered by the treatments imposed on soybean genotype TGx2001-25DM, bacterial inoculation on its own markedly increased shoot Fe levels in PAN 1614 and TGx1830-20E when compared to pure NO_3_^−^ feeding or inoculated + NO_3_^−^ (Fig. 4a). The former and latter treatments accumulated similar levels of Fe in the shoots of TGx1830-20E. Shoot Zn concentrations were also altered by the imposed treatments, with *Bradyrhizobium* application markedly increasing shoot Zn levels over pure NO_3_^−^ feeding and inoculated + NO_3_^−^ in PAN 1614 and TGx2001-25DM (Fig. 4b), but the inoculated + NO_3_^−^ treatment increased Zn accumulation over bacterial inoculation and NO_3_^−^ feeding in TGx1830-20E and over pure NO_3_^−^ feeding in TGx2001-25DM (Fig. 4b). Shoot Mn concentration rose significantly in all three soybean genotypes with bacterial inoculation when compared to pure NO_3_^−^ feeding or inoculated + NO_3_^−^ (Fig. 4c). Pure NO_3_^−^ feeding significantly increased shoot Cu levels in TGx1830-20E. Similar concentrations of Cu in PAN 1614 were accumulated in pure NO_3_^−^ feeding and inoculated + NO_3_^−^ plants but the latter treatment produced greater levels of Cu in TGx2001-25DM. The lowest concentration of Cu in all genotypes was recorded in purely inoculated plants (Fig. 4d).

**Fig. 5:**
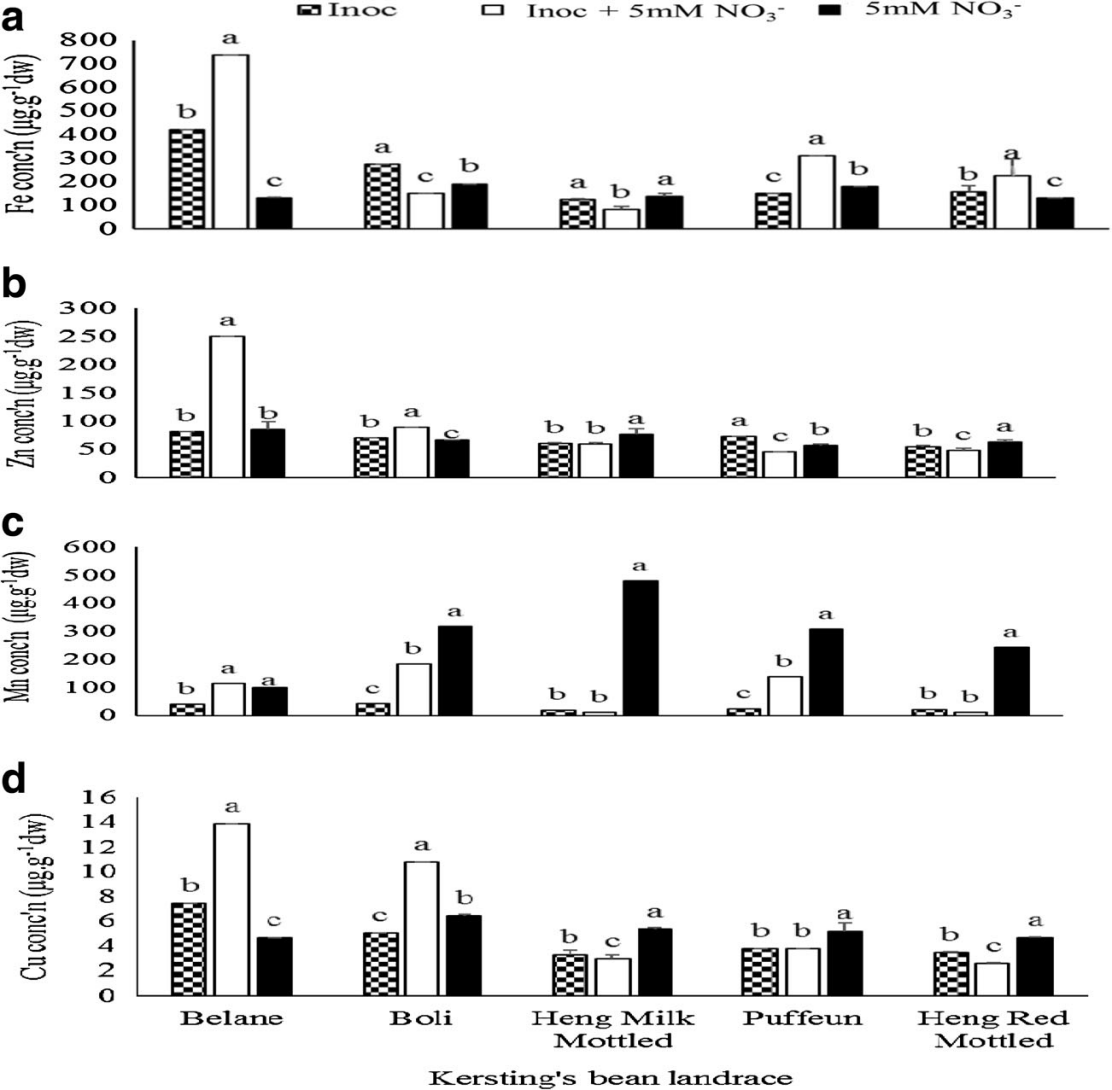
Landrace x treatment interaction on **a** Fe **b** Zn **c** Mn and **d** Cu concentration in shoots of Kersting’s groundnut landraces grown in the glasshouse

The Kersting’s groundnut landraces markedly differed in the concentration of Fe, Zn, Mn and Cu in shoots when planted with the imposed treatments. The inoculated + NO_3_^−^ treatment increased the levels of Fe in the Belane, Puffeun and Heng Red Mottled landraces relative to the other treatments (Fig. 5a). Sole *Bradyrhizobium* inoculation increased the concentration of Fe in the Boli landrace and together with the pure NO_3_^−^ feeding treatment, increased the level of Fe in the Heng Milk Mottled landrace (Fig. 5a). Zn concentrations increased significantly with the inoculated + NO_3_^−^ treatment in the Belane and Boli landraces, while the Zn concentration of Heng Milk Mottled and Heng Red Mottled landraces were increased with pure NO_3_^−^ feeding (Fig. 5b). Pure NO_3_^-^ feeding increased Mn concentration in all five landraces (Fig. 5c). Inoculation alone however increased the Zn concentration in Puffeun landrace. Levels of Cu in shoots of the test landraces were increased by the inoculated + NO_3_^−^ treatment in the Belane and Boli landraces (Fig. 5d). However, pure NO_3_^−^ feeding increased Cu concentration in shoots of Heng Milk Mottled, Puffeun and Heng Red Mottled landraces (Fig. 5d).

**Fig. 6:**
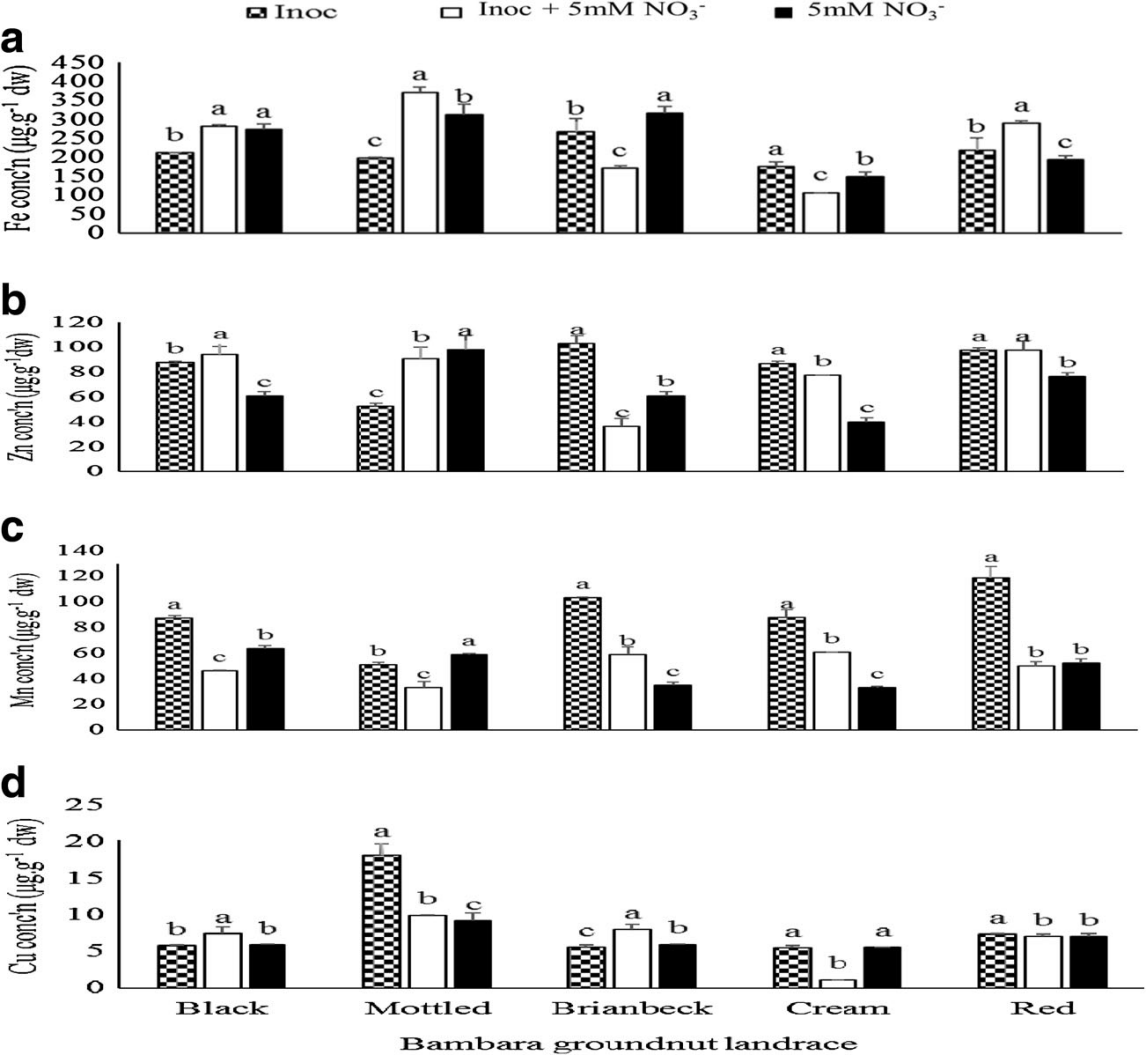
Genotype x treatment interaction on **a** Fe **b** Zn and **c** Mn **d** Cu concentration in shoots of Bambara landraces grown in the glasshouse

Bambara groundnut landraces differed in their individual responses to the three experimental treatments. As shown in Fig. 6a, the inoculated + NO_3_^−^ treatment markedly increased shoot Fe in the Black, Mottled and Red landraces of Bambara landraces relative to pure NO_3_^−^ feeding and bacterial inoculation alone. However pure NO_3_^−^ feeding also significantly caused greater accumulation of Fe in shoots of Black and Brianbeck landraces. *Bradyrhizobium* inoculation alone markedly increased Zn concentration in Brianbeck and Cream landraces, and together with the inoculated + NO_3_^−^ treatment also significantly increased shoot Zn levels in the Red landrace (Fig. 6b). It was in only Mottled landrace that pure NO_3_^−^ feeding induced the highest shoot accumulation of Zn (Fig. 6b). Shoot Mn concentration showed a strong response to *Bradyrhizobium* inoculation as it elicited a marked accumulation of this trace element in the Black, Brianbeck, Cream and Red landraces when compared to the other two treatments (Fig. 6c). Bacterial inoculation also significantly raised the concentration of Cu in the Mottled, Cream and Red landraces, while inoculated + NO_3_^−^ treatment induced much greater Cu level in Black and Brianbeck landraces (Fig. 6d).

### Correlation analysis

Correlation analysis revealed significant relationships between shoot mineral concentrations and symbiotic parameters such as shoot dry matter, nodule dry weights and nodule number in the test legumes. For example, we found significant correlations between shoot biomass of soybean genotype PAN1614 and P (*r* = 0.86**), S (*r* = 0.89**), Fe (*r* = 0.78*), Zn (*r* = 0.82**) and Mn (*r* = 0.83), just as its nodule dry matter was also significantly correlated with P (*r* = 0.70*), K (*r* = 0.90**), S (*r* = 0.72***), Fe (*r* = 0.95***), Zn (0.95***) and Mn (*r* = 0.92**). PAN1614 nodule number also correlated with Fe (*r* = 0.80*), Zn (r = 0.70*) and Mn (*r* = 0.76*). Nodule dry weights of Kersting’s groundnut were significantly correlated with P (r=0.99***), just as nodule number was markedly correlated with Ca (*r* = 0.81**), Fe (r = 0.82**), and Cu (r = 0.70*). The results for Bambara groundnut also showed a pattern of significant correlations between shoot mineral concentrations and symbiotic parameters of landraces. For example, nodule number of the Black Bambara groundnut landrace correlated significantly with shoot P (r = 0.82**), S (*r* = 0.73*) and Zn (r = 0.83**)**), in the same manner that nodule dry weights correlated with P (*r* = 0.87**) and S (*r* = 0.91*). Nodule dry mater of Brianbeck correlated with P (r = 0.7*), Ca (*r* = 0.0.82**) and Mn (*r* = 0.94***). The Cream landrace of Bambara groundnut also showed significantly marked correlation with P (*r* = 0.68*), Ca (r = 0.86**), Zn (r = 0.92**) and Mn (*r* = 0.98***). Likewise, nodule number correlated with Ca (r = 0.95***), Zn (*r* = 0.0.97***) and Mn (*r* = 0.97***). The shoot dry matter of Red landrace significantly correlated with K (r = 0.78*), Fe (*r* = 0.74*) and Zn (r = 0.98***), while nodule dry matter also significantly correlated with P (*r* = 0.84**), Ca (r = 0.90**), Zn (*r* = 0.0.73*) and Mn (r = 0.78*).

## Discussion

N_2_ fixation is the source of N for meeting the N demand of nodulated legumes. This process can differ between and among legume species and genotypes. In this study, we found differences in N_2_ fixation between three soybean genotypes as well as among five Kersting’s bean and Bambara groundnut landraces treated to 5 mM NO_3_^−^ and/or rhizobial inoculation (Tables 1, 2 and 3). Here, the three test soybean genotypes responded differently to *Bradyrhizobium* inoculation, NO_3_^−^ feeding and the combined application of NO_3_^−^ plus bacterial inoculation. Soybean genotypes PAN 1614 and TGx2001-25DM exhibited better plant growth (measured as shoot biomass) with inoculation as a result of higher nodulation and N_2_ fixation (Table 1). Increased nodulation and plant growth were also observed in the Kersting’s bean and Bambara groundnut landraces with inoculation (Tables 2 and 3).

However, soybean, Kersting’s bean and Bambara groundnut symbioses revealed some sensitivity to NO_3_^−^ nutrition. As shown in Tables 1, 2 and 3, NO_3_^−^ inhibition of nodule formation significantly reduced nodule numbers from 74 to 54, and nodule mass from 230 to 70 mg.plant^- 1^ in soybean. In Kersting’s groundnut, nodule numbers were reduced from 47 to 32 and nodule mass from 90 to 20 mg. plant^−1^. Similarly, NO_3_^−^ inhibition of nodulation decreased nodule number from 41 to 34 per plant and nodule dry weight from 210 to 90 mg. plant^−1^ in Bambara groundnut. As shown in Figs. 1, 2 and 3, nodule dry matter was reduced by more than 5% in each of the soybean, Kersting’s and Bambara genotypes/landraces. However, some genotypes and landraces appeared to be more affected by NO_3_^−^ inhibition of nodule formation than the others, a finding consistent with the report that some landraces of Kersting’s bean and Bambara groundnut are NO_3_^−^ tolerant (Dakora et al. 1992; Dakora 1998).

We found some subtle effects of NO_3_^−^ on the symbioses of the three legume species. As shown in Fig. 1, nodule mass was generally more sensitive to NO_3_^−^ than nodule number for the three soybean genotypes. Similarly, nodule mass of Kersting’s bean landraces Boli, Heng Milk Mottled and Puffeun was also more sensitive to NO_3_^−^ inhibition than nodule numbers (Fig. 2). Although the results of Bambara groundnut were similar to those of Kerting;s bean, the effect of NO_3_^−^ on the symbiosis was less pronounced, a clear indication of tolerance of the species to added NO_3_^−^ when compared to soybean (Dakora et al. 1992; Dakora 1998).

Ecologically speaking, legumes adapted to growth in nutrient-poor soils tend to underperform with mineral fertilization [e.g. *Cyclopia longifolia* (Vogel L.)] and usually prefer rhizobial symbiosis as N source and therefore fix more N_2_ when nodulated by effective rhizobial strains (Muofhe and Dakora 1999; Maseko and Dakora 2015). Such legumes tend to perform poorly when grown with N and other mineral fertilizers (Ayisi et al. 2000; Mndzebele and Dakora 2017). In contrast, legumes that are adapted to moderately N-rich soils seem capable of depending jointly on symbiosis and mineral N for their nutrition.

In this study, ICP-MS analysis of plant shoots revealed marked differences in micronutrient levels in response to the imposed N sources. Compared to inoculated + NO_3_^−^ treatment, *Bradyrhizobium* inoculation alone significantly increased Fe concentration in PAN 1614 and TGx1830-20E, Zn concentration in PAN 1614 and TGx2001-25DM, as well as Mn in PAN 1614, TGx1830-20E and TGx2001-25DM (Fig. 4). Coincidentally, *Bradyrhizobium* application also markedly increased symbiotic performance (i.e. shoot biomass, nodule number and nodule dry matter per plant) in the same soybean genotypes (Fig. 1). With Kersting’s groundnut, increased plant growth as a result of *Bradyrhizobium* inoculation (Fig. 2) did not translate into increased levels of mineral nutrients in shoots of the same plants. However, there was an increase in the concentration of Fe, Zn and Cu in the landrace Belane when inoculated + 5 mM NO_3_^−^. The same treatment also increased the concentration of Zn and Cu in Boli landrace, while pure NO_3_^−^ -feeding increased Fe, Zn and Cu in Heng Milk Mottled landrace, only Zn and Cu in the Heng Red Mottled landrace, and increased Mn concentration in all five landraces (Fig. 5).

The micronutrient data for Bambara groundnut showed a similar pattern and relationship with symbiotic performance as Kersting’s bean. Here, *Bradyrhizobium* inoculation markedly increased the concentration of Zn in Brianbeck, Cream and Red landraces, Mn in Black, Brianbeck, Cream and Red landraces, and Cu in Mottled, Cream and Red landraces when compared to inoculated + NO_3_^−^ treatment (Fig. 6). In contrast, Fe concentration was significantly greater in the inoculated + NO_3_^−^ treatment of the Black, Mottled and Red Bambara groundnut landraces (Fig. 6a). Again, these significant increases in shoot micronutrient accumulation coincided with greater symbiotic performance of the genotypes/landraces that were treated to *Bradyrhizobium* or inoculated + NO_3_^−^.

It has been shown that accumulation of micronutrients such as Fe, Zn, Mn and Cu in nodulated cowpea is linked to symbiotic efficiency, and is greater where there is higher N_2_-fixing efficiency in root nodules (Belane et al. 2014). The micronutrient data obtained in this study can therefore be interpreted to mean that NO_3_^−^ inhibition of N_2_ fixation in soybean and Bambara groundnut altered trace element accumulation in plant shoots. This could be attributed to the suppressive effect of NO_3_^−^ on nodulation and N_2_ fixation (Escuredo et al. 1996; Streeter and Wong 1988). It was therefore not surprising that where *Bradyrhizobium* inoculation increased symbiotic performance, micronutrient levels were also increased in shoots, and where symbiotic performance was low in inoculated + NO_3_^−^ treatment, trace element concentrations were correspondingly low. However, this trend was not observed in Kersting’s groundnut landraces, as greater mineral concentrations were recorded in the inoculated + NO_3_^−^ plants, followed by 5 mM NO_3_^−^ fed plants, despite having much higher plant growth when seedlings were solely dependent on bacterial inoculation. Decreasing N_2_ fixation by means of NO_3_^−^ application to nodulated plants decreased micronutrient accumulation in some of the test legume genotypes/landraces. This therefore suggests that factors that inhibit N_2_ fixation in nodulated legumes (e.g. pO_2_, NO_3_^−^, waterlogging, drought, etc.) can potentially reduce trace element concentrations in plant organs.

Although it can be argued that NO_3_^−^-fed and purely symbiotic nodulated legumes may have different physiologies, in this study symbiotic parameters (e.g. shoot biomass, nodule number and nodule dry weights) were significantly correlated with shoot concentration of both macronutrients (P, K, Ca, Mg and S) and micronutrients (Fe, Zn, Mn and Cu). For example, we found significant correlations between shoot biomass of soybean genotype PAN1614 and P (*r* = 0.86**), S (*r* = 0.89**), Fe (*r* = 0.78*), Zn (*r* = 0.82**) and Mn (*r* = 0.83), just as its nodule dry matter was also significantly correlated with P (*r* = 0.70*), K (*r* = 0.90**), S (*r* = 0.72***), Fe (*r* = 0.95***), Zn (0.95***) and Mn (*r* = 0.92**). We therefore interpret these results to mean that NO_3_^−^ inhibition of |nodulation and N_2_ fixation was the main cause of the decreased micronutrient accumulation. In Africa, the soils are low in micronutrients, as a result about 263 million people are suffering from micronutrient deficiency. Therefore, any agronomic practice that further reduces the levels of trace elements in plant shoots is likely to heighten micronutrient deficiency among rural Africans. Although only the grain of the three legumes used in this study is eaten; in the case of cowpea both the leaves and grain are consumed as food. So, reductions in trace element concentration of cowpea shoots would no doubt promote micronutrient deficiency in rural children. It would also be interesting to determine whether these findings apply to non-legumes.

Taken together, this study has demonstrated significant NO_3_^−^ inhibition of nodule formation and N_2_ fixation in soybean and Bambara groundnut, but to a lesser extent in Kersting’s groundnut. The data have also shown preferences for N sources (NO_3_^−^, inoculated + NO_3_^−^ and purely symbiotic) by the genotypes and landraces of the three legume species tested. Furthermore, micronutrient concentrations in plant shoots varied with genotype/landrace and increased with N_2_-fixing efficiency in soybean and Bambara groundnut. Decreasing N_2_ fixation with NO_3_^−^ application reduced trace element accumulation in shoots of soybean and Bambara groundnut, but not Kersting’s groundnut.
